# The Endocannabinoid System: A Bridge between Alzheimer’s Disease and Gut Microbiota

**DOI:** 10.3390/life11090934

**Published:** 2021-09-08

**Authors:** Tiziana Bisogno, Anna Lauritano, Fabiana Piscitelli

**Affiliations:** 1 Endocannabinoid Research Group, Istituto di Farmacologia Traslazionale, Consiglio Nazionale Delle Ricerche, Area Della Ricerca di Roma 2 Via Fosso del Cavaliere 100, 00133 Roma, Italy; 2 Endocannabinoid Research Group, Istituto di Chimica Biomolecolare, Via Campi Flegrei 34, 80078 Pozzuoli, Italy; anna.lauritano@icb.cnr.it

**Keywords:** endocannabinoid system, microbiota, Alzheimer’s disease, gut-brain axis

## Abstract

Alzheimer’s disease (AD) is a neurodegenerative disease that progresses from mild cognitive impairment to severe dementia over time. The main clinical hallmarks of the disease (e.g., beta-amyloid plaques and neurofibrillary tangles) begin during preclinical AD when cognitive deficits are not yet apparent. Hence, a more profound understanding of AD pathogenesis is needed to develop new therapeutic strategies. In this context, the endocannabinoid (eCB) system and the gut microbiome are increasingly emerging as important players in maintaining the general homeostasis and the health status of the host. However, their interaction has come to light just recently with gut microbiota regulating the eCB tone at both receptor and enzyme levels in intestinal and adipose tissues. Importantly, eCB system and gut microbiome, have been suggested to play a role in AD in both animal and human studies. Therefore, the microbiome gut-brain axis and the eCB system are potential common denominators in the AD physiopathology. Hence, the aim of this review is to provide a general overview on the role of both the eCB system and the microbiome gut-brain axis in AD and to suggest possible mechanisms that underlie the potential interplay of these two systems.

## 1. Introduction

Alzheimer’s disease (AD) is a chronic age-related progressive neurodegenerative disorder accounting for ~80% of dementia globally. Despite decades of intensive research, AD remains incurable and is still a challenge for global health. Indeed, current therapies provide only symptomatic relief and promising preclinical results have repeatedly failed in phase III clinical trials. Thus, the full understanding of the disease pathogenesis and the identification of therapeutic strategies that may prevent or delay disease progression appear urgent [1].

In this scenario, increasing interest has been focused on the gut microbiome and the endocannabinoid (eCB) system as emerging targets involved in the control of AD.

The community of bacteria, yeast, archaea, viruses, protozoa, and parasite that inhabit human gastrointestinal (GI) tract, collectively known as microbiota, undergoes dynamic changes throughout life [2]. An increasing number of studies have explored a possible bidirectional connection between gut microbiota and the brain, for which the term gut-brain axis has been coined [3,4]. Moreover, the link between the gut microbiota and human diseases is more and more evident as alterations in gut microbial community composition have been reported in several pathological conditions including neurological and autoimmune disorders, obesity and cancer [5,6,7,8,9,10]. The microbiome studies on a transgenic AD model and patients highlighted alterations in gut microbiota providing new understanding of AD and additional features for its pathological characterization [11,12]. On the other hand, the eCB system that is ubiquitously expressed throughout the gut, periphery and brain is now well recognized to participate in almost all human physiological processes and to be involved in several pathological conditions [13,14,15,16].

Studies performed in both AD-like animal models and patients suggested that eCB system alterations are associated with AD pathophysiology, and that its pharmacological modulation may have disease-modifying effects [17,18]. In the last few years, the interconnection of the eCB system and the gut microbiota has been addressed and different aspects of regulation and dysregulation of the gut microbiome and eCB system mainly in obesity and metabolic disorders excellently reviewed [19,20]. Recently, the eCB system and the gut microbiota dysfunctions were also reported in neuropsychiatric disorders [21,22].

In this review, we first provide an overview of the microbiota gut-brain axis and the eCB system, then we consider the crosstalk between the gut microbiota and the eCB system or AD physiopathology. Finally, we discuss the potential connection between the eCB system and the gut-brain axis in AD.

## 2. The eCB System and the Endocannabinoidome

**Figure 1:**
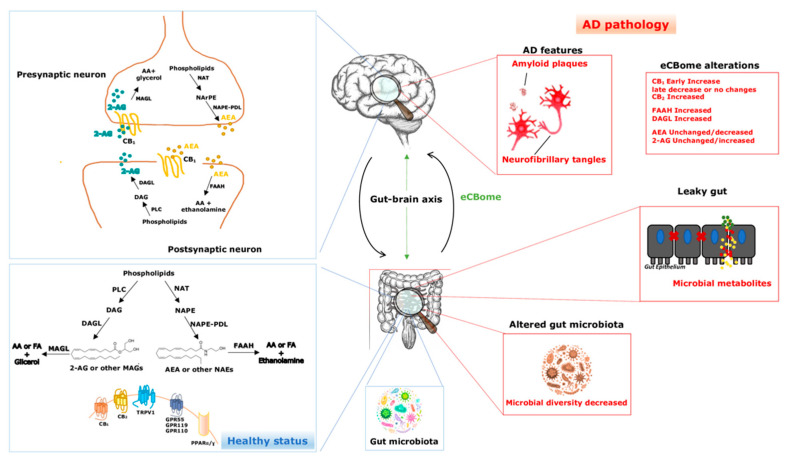
The endocannabinoidome and the gut-brain axis in physiological conditions and in Alzheimer’s disease. Blue lines indicate a healthy status, though red lines indicate AD pathology. Green arrows denote the bidirectional interplay between the eCBome and the gut-brain axis. eCBome—endocannabinoidome; AD—Alzheimer’s disease; AEA—anandamide; 2-AG—2-arachidonoylglycerol; NAEs—*N*-acylethanolamines; MAGs—monoacylglycerols; AA—arachidonic acid; FA—fatty acids; PLC—phospholipase C; DAGL—diacylglycerol lipase; MAGL—monoacylglycerol lipase; NArPE—*N*-arachidonoyl-phosphatidylethanolamine; NAPE—*N*-acyl-phosphatidylethanolamine; NAPE-PLD—NAPE-specific phospholipase D-like enzyme; NAT—*N*-acyl-transferase; FAAH—fatty acid amide hydrolase; CB_1_ and CB_2_—cannabinoid receptor type-1 and 2; TRPV1—transient receptor potential cation channels, such as the vanilloid type-1; GPR55, GPR119 and GPR110 G—protein-coupled receptor 55, 119 and 110; PPARα and PPARγ—peroxisome proliferator-activated receptor-α and -γ.

The eCB system has classically been described as a complex pleiotropic system comprised of two cannabinoid responsive G-protein-coupled receptors referred as the eCB receptor type-1 and type-2 (CB_1_ and CB_2_), endogenous lipid-derived ligands for such receptors, known as eCBs, and at least five enzymes responsible for eCB biosynthesis and degradation [23]. The two most studied eCBs are *N*-arachidonoylethanolamine (AEA), also known as anandamide, and 2-arachidonoylglycerol (2-AG) which belong to the *N*-acylethanolamine (NAEs) and 2-monoacylglycerol (2-MAGs) families, respectively [24] (Figure 1). Unlike classical neurotransmitters, or many other intracellular signaling molecules, which are stored in vesicles before to be released, AEA and 2-AG are synthesized “on demand” from arachidonic acid (AA) in a cell and time-specific manner through enzymatic activation by multiple pathways in membrane of different cell types such as neurons, adipocytes, and skeletal muscle cells, in response to increased intracellular Ca^2+^ concentration, membrane depolarization, and/or receptor stimulation [25,26]. In brief, AEA is biosynthesized from the hydrolysis of *N*-acyl-phosphatidylethanolamines (NAPE) by NAPE-specific phospholipase D-like enzyme (NAPE-PLD) or via other routes not involving NAPE-PLD [27]. On the other hand, 2-AG is produced from the hydrolysis of diacylglycerol (DAG), by either DAG lipase-α or -β (DAGL-α or -β), although most 2-AG mediating synaptic transmission in adult brain is generated mostly by DAGL-α [28,29]. However, AEA and 2-AG are inactivated in respective target tissues differently. Indeed, AEA is hydrolyzed to AA and ethanolamine by fatty acid amide hydrolase (FAAH), whereas 2-AG is mostly hydrolyzed by MAG lipase (MAGL) into AA and glycerol [30,31]. It is well recognized that eCBs modulate retrograde signaling in the brain, providing a mechanism for inhibitory feedback to regulate neurotransmitter release [32,33]. This unique function of eCBs suggested their investigation as therapeutic targets for human diseases affecting central nervous system (CNS) [13]. Indeed, the eCB system dysregulation in the CNS has been increasingly implicated in the physiopathology of neurodegenerative and neuropsychiatric disorders, such as AD [18], Parkinson’s disease [34], Huntington’s disease [35], multiple sclerosis [36] schizophrenia [37] and anxiety disorders [38].

CB_1_ and CB_2_ are differently expressed throughout human body and their distribution correlated with their specific physiological roles. The CB_1_ is the most abundant and widespread GPCR in the mammalian CNS but it is also expressed in non-neuronal peripheral tissues, including the gut, where it is involved in the nociception, adipogenesis and pro-inflammation processes [39]. On the other hand, CB_2_ is prevalent in the periphery, particularly within immune cells including microglia, but its expression in neurons is still enigmatic [38,40]. CB_2_ activation reduces inflammatory mediator release and it seems to be the main target for inflammation-dependent neurodegeneration [41]. Interestingly, CB_1_ and CB_2_ are not the only receptors whose activity are responsive to AEA and 2-AG, as both ligands are able to modulate other GPCRs such as GPR-18, GPR-55 and GPR-119, the thermosensitive transient receptor potential cation channels, such as the vanilloid type-1 (TRPV1), as well as peroxisome proliferator-activated receptor-α and -γ (PPAR-α and -γ) [39]. The identification of a continuous increasing number of bioactive long chain fatty acid amides having eCB-like properties, such as other NAEs, including *N*-palmitoylethanolamine (PEA) and *N*-oleylethanolamine (OEA) or *N*-acyl amino acids/dopamine/taurine/serotonines, has allowed to expand the concept of the eCB system towards the “endocannabinoidome” (eCBome) [42]. This new plethora of bioactive compounds share with eCBs metabolic pathways and molecular targets, not only CB_1_ and CB_2_ [20,43], but also GPR-55, TRPV1 and PPAR [44,45] mimicking the physiological features of eCBs.

## 3. Microbiota Gut-Brain Axis: Background

### 3.1. Gut Microbiome

In the past few decades, the scientific community has increased its interest in the microbiome and how it may impact our daily lives. Human microbiota accounts for trillions of microorganisms that live in symbiotic balance with us [46] and is a key player of both host physiology and human health and disease. In the last decades, technological advances in sequencing methodologies and in bioinformatic tools have allowed to make great strides in terms of microbiota composition with cheaper costs than in the past. Indeed, over 10 million of genes in our body are microbial, resulting >99% of our total genes [46,47,48,49,50,51,52]. Gut, skin, oral cavities, eyes and the urogenital tract are the main human sites colonized by microbes. Among the others, the gut microbiota is the best known and the most studied, and its full investigation is helping to understand the microbiome role in many physiological and pathological conditions and to develop new therapeutic strategies. The GI tract is a very hospitable ground for many microbes, providing an energy rich, anaerobic environment in which the microbes can thrive, including not only bacteria, which are the most well characterized, but also yeasts, archaea, parasites such as helminths, viruses, and protozoa [53,54,55,56,57]. In this favorable environment, microbes contribute to host metabolism, protection and immune development and maintenance. Data obtained from the Human Microbiome Project and MetaHIT show that the microbiome is classified into 11 different phyla with Proteobacteria, Firmicutes, Actinobacteria and Bacteroidetes [58,59,60] accounting for the 90% of the total microbiome, whereas Fusobacteria and Verrucomicrobia phyla are less abundant [53].

In terms of evolution, the microbiota has been a constant companion throughout our history, living in symbiotic balance with our body. Indeed, while our genome is stable during lifetime, the microbiome is dynamic [61], different in terms of composition and abundance [62,63] and affected by external inputs. After birth specific taxa are hosted in our body with high abundance of Enterobacteriaceae, Bifidobacteriaceae, and Clostridiaceae families, and low abundance of Lachnospiraceae and Ruminococcaceae [64,65,66]. As the baby grows the diversity in microbial families composition increases with a majority of anaerobic taxa [67,68,69] with a specific enrichment in pathways supporting ongoing development, which are functional for the healthy state [70]. The microbiota continues to change until adulthood and remains stable until old age if unperturbed by pathological or environmental conditions [2]. Indeed, the gut microbiota composition in the elderly decreases, and this event correlates with some age-related impairments in humans, such as frailty and cognitive deficits [71,72,73]. On the other hand, animal studies have shown that in old mice the diversity of microbiota composition increased in comparison to younger rodents [74]. Moreover, some studies have shown that Bacteroides/Prevotella, Eubacteriumrectale/Clostridium coccoides, and Ruminococcus prevailed in the microbiota of people aged between 70 and 100 years [75] and studies in semi-supercentenarians, from 105 to 109 years of age, showed a less abundance of Akkermansia, suggesting a specific role of the gut microbiota composition change in promoting longevity and health [76,77].

### 3.2. Gut-Brain Axis Bidirectional Interplay

The CNS and the GI are in constant communication through a bidirectional pathway, the so-called “gut-brain axis” [78,79]. This pathway integrates neural, hormonal and immunological signals that provide CNS regulation of permeability, secretion, motility, and immunity of the digestive tract [80]. The gut microbiome can affect brain functions by influencing the host metabolism and through the synthesis of biological active mediators, which in turn activate their molecular targets expressed on the afferent fibers of the vagus nerve (VN) and reduce the gut and blood-brain barrier (BBB) permeability, block the microglia and astrocyte activation triggering the gut and brain homeostasis [81,82]. The autonomic nervous system (ANS), immune system and the hypothalamic-pituitary-adrenal (HPA) axis allow the communication between gut and brain. Moreover, they can also communicate by the production of neurotransmitters and short chain fatty acids (SCFAs) [81,82].

The ANS comprises of three branches: the sympathetic, parasympathetic, and the enteric nervous system (ENS) [82], through which control GI functions [83]. In particular, the ANS drives both afferent and efferent neural signals between the gut and the brain, respectively. The ENS is located in the gut lignin and regulates mainly motor functions and secretion of the GI tract. Sympathetic and parasympathetic system communicate with CNS via prevertebral ganglia and VN, respectively. The gut microbiota produces several neuromodulators, that when released, influence CNS activities interacting with the host neurons through the afferent pathways from ENS and ANS, both locally and centrally [84]. The HPA axis is the main non-neuronal pathways within the bidirectional communication between the gut microbiome and the brain. It is a neuroendocrine coordinator of the adaptive responses against stress by releasing corticotrophin releasing factor (CRF) from the paraventricular nucleus (PVN) of the hypothalamus [83]. Interestingly, there is interplay between the VN and the HPA axis as showed by Hosoi et al. [85], via release of IL-1β. Recent studies have shown the interaction of the gut microbiota with the CNS resident immune system, i.e., microglial cells, which are considered the macrophages of the brain and play a key role in the homeostasis and development of the brain [86]. Germ-free (GF) mice, which are totally devoid of bacteria, displayed global defects in microglia, which was restored by a reconstitution of the intestinal microbiota or by supplementation with SCFAs [86]. Moreover, monocyte migration induced by tumor necrosis factor-alpha (TNF-α) release by activated microglia was reverted by probiotic treatment improving inflammation-associated sickness behavior [87,88]. The gut microbiota has been shown to synthesize and respond to host neuroendocrine signaling molecules, including catecholamines, GABA, histamine, serotonin, tryptophan and kynurenine [89] which are deeply involved in host mood and cognition. Several bacterial species, e.g., *Escherichia* and *Bacillus* produced catecholamines, while other such as *Lactobacillus* synthesized GABA [82]. Serotonin (5-HT) is released by enterochomaffin cells (ECs), synthesized starting from dietary tryptophan. Although studies in GF mice have demonstrated that specific gut bacterial species increase colonic 5-HT levels, via tryptophan hydroxylase-1 (TPH1) overexpression [90,91], the impossibility of 5-HT to cross the BBB suggests that variation in intestinal 5-HT levels are unlikely to produce direct effect on the brain [89,92]. However, 5-HT released by ECs may affect the gut-brain axis by modulating the gut vagal afferent activity and inflammatory responses [89]. The gut microbiota is able to control host tryptophan metabolism along the kynurenine pathway, thereby decreasing serotonin synthesis, which in turn can have an effect on disturbances associated with serotonergic neurotransmission [93]. Indeed, it has been shown kynurenine and its metabolites are implicated in mental health [94]. Interestingly, some bacterial strains are able to synthesize histamine which plays a key role in host physiology, including the regulation of immune functions and wakefulness [89]. Therefore, it seems to be well established histamine role in the regulation of the interplay host-microbe as a neuroendocrine-immune mediator [82].

SCFAs (mostly acetate, propionate and butyrate) are the most studied gut microbial-derived metabolites and have a fundamental role in host physiology [95,96,97]. It has been shown that SCFAs gastrointestinal levels are associated with CNS disorders, such as AD or autism spectrum disorder (ASD), demonstrating their key role in the gut-brain communication [98,99]. SCFAs production is regulated by many different host, environmental, dietary and microbiological factors and their primary source is the microbial fermentation of specific host-indigestible dietary fibers [100]. SCFAs have been detected in human feces and in hepatocytes but only acetate has been reported to be present also in the cerebral spinal fluid (CSF) [89]. Growing body of evidence shows that gut microbial-derived SCFAs might be able to direct affect host CNS physiology. Once produced in the gut, SCFAs can therefore cross the BBB via the circulation system and influence the microglia [101]. The presence of propionate or butyrate in the brain has never been reported, however, propionate can cross the BBB and its molecular target (free fatty acid receptor-3, FFAR3) is expressed in human brain endothelium [102]. Moreover, propionate has also been detected in human saliva and its levels correlated with dementia [103] and AD [104].

## 4. The Pathogenic Role of Gut Microbiota in AD

AD is a fatal neurodegenerative disorder characterized by a progressive memory loss and cognitive impairment [105]. The cardinal pathological features of the AD brain are the presence of neurofibrillary tangles, intracellular lesions due to hyperphosphorylated tau protein, and senile neuritic plaques consisting of extracellular insoluble forms of amyloid-β-peptide (Aβ). The latter is produced through the sequential cleavage of amyloid precursor protein (APP) by β-secretase β-site APP cleavage enzyme 1, and the γ-secretase complex [105]. The full understanding of the pathogenesis of AD is still challenging and many hypotheses including neuroinflammation, oxidative stress, mitochondrial dysfunction, lipid metabolism, apoptosis, calcium and metal dyshomeostasis, and epigenetic changes have been proposed [106,107,108,109]. In addition, infectious agents have been found in the brain and postulated to be, through multiple mechanisms, involved in the pathogenesis of AD. In this regard, the periodontitis-associated pathogen *Porphyromonas gingivalis* might affect the development and/or progression of AD and therapeutic approaches aimed at counteract both periodontitis and AD are under investigation [110,111]. None of the current theories explain all the histopathological and multifactorial molecular changes described in AD and although tremendous efforts have been made for the treatment of AD, no efficient disease-modifying therapeutics are available. In the recent years, the microbiota-gut-brain axis has emerged as a potential therapeutic target for the treatment of several pathological conditions including, among the other, brain disorders [112], metabolic diseases [113,114] and inflammatory bowel disease [115]. Relevant preclinical and epidemiological studies have associated the common intestinal disorders constipation, diarrhea, irritable bowel syndrome (IBS), inflammatory bowel disease (IBD) and intestinal bacterial overgrowth to the increased risk in developing dementia or AD [116,117,118]. Several reports indicate that gut microbiota composition and activity affect the pathogenesis of AD through many pathways, including neurotransmitters, metabolites and chronic neuroinflammation. Some bacteria metabolize or produce a broad range of neurotransmitters including dopamine, 5-HT, α-aminobutyric acid (GABA) and acetylcholine [119,120,121,122], and even though they cannot cross the protective BBB, they might affect the physiological events in the brain through the VN and its afferent neurons [123]. As above reported, gut microbiota control the metabolism of the amino acid tryptophan and can decrease 5-HT availability by enhancing the pool of tryptophan available for kynurenine synthesis [93]. Acetylcholine, produced by *Bacillus subtilis* and *Lactobacillus plantarum*, can reduce the production of interleukin-6 and 1β (IL-6, IL-1β) and TNF-α, while dopamine, produced by *Bacillus* and *Escherichia*, modulates NLRP3 (nucleotide-binding oligomerization domain (NOD)-, leucine-rich repeat (LRR)-, and pyrin domain (PYD)-containing protein 3) inflammasome degradation and exerts anti-inflammatory or pro-inflammatory functions via activation of the dopamine receptors D1, D2 and D3, respectively [124,125,126]. Microbiota might modulate inhibitory/excitatory neurotransmission in CNS as *Bifidobacterium* species metabolize glutamate to produce GABA, GF mice exhibited altered *N*-methyl-D-aspartate (NMDA) receptor expression and increased glutamatergic activity and, reduction of GABA and enhancement of glutamate levels were reported in AβPPswe-PS1dE9 mice [127,128,129]. SCFAs are capable of potently inhibit Aβ aggregations in vitro, modulating maturation and function of microglia in the brain [86,130] and butyric acid may provide therapeutic benefits for AD through epigenetic mechanisms [131]. Primary bile acids, post-prandial secreted into the intestine, are further metabolized by the action of the gut microbiome into secondary bile acids that promote protein misfolding and impaired intra-cellular metabolism [132]. In addition, an alteration of bile acid profile in AD patients was associated with impairment of cognitive functions and changes in CSF markers of disease [133]. The microbial-derived metabolite trimethylamine *N*-oxide (TMAO) increases β-secretase activity and Aβ accumulation, and plasma TMAO levels were associated with deteriorative cognitive functions and AD pathology in APP/PS1 mice [134]. Gut bacteria such as *E. coli*, *Salmonella* and *Citrobacter* produce amyloids (curli, tau, Aβ, α-syn, and prion) that promote misfolding of Aβ oligomers and fibrils, contributing to AD pathology [135]. In addition, the activation of toll-like receptors 2/1 (TLR 2/1), cluster of differentiation 14 (CD14), and nuclear factor-κB (NF-κB) promotes release of pro-inflammatory cytokines that by initiating immunogenic reactions contribute to neurodegeneration [136,137]. Bacterial endotoxins, found within the typical senile plaque lesions of the AD brains, exert a key role in the inflammatory processes associated with amyloidosis and AD [138]. Of note, increased pro-inflammatory and reduced anti-inflammatory cytokine blood levels were detected in patients with cognitive impairment and brain amyloidosis. The peripheral inflammatory state of patients was also associated with the increase of a pro-inflammatory *Escherichia*/*Shigella* and reduction of *E. rectale* bacteria abundance [139].

The link between gut microbiota and its dysbiosis as a risk factor for AD has supported by research of probiotics with anti-AD potential (Table 1) as well as by studies investigating the efficacy of fecal microbial transplantation (FMT) as a new therapeutic approach to treat dementia and AD.

In Aβ (1–42) injected rats, probiotics intake (*Lactobacillus acidophilus*, *L. fermentum*, *Bifidobacterium lactis*, and *B. longum*) for 8 weeks prevented learning and memory impairment and decreased the number and size of plaques [140]. In the same model, *Lactobacillus acidophilus*, *Bifidobacterium bifidum* and *Bifidobacterium longum* restored the hippocampus dependent spatial memory and synaptic plasticity damaged after Aβ administration [141]. The administration of SLAB51, a novel formulation of *lactic acid bacteria* and *bifido bacteria*, to a triple-transgenic mouse model of AD (3xTg-AD) in the early stages of the disease counteracted cognitive decline and brain damage, modified intestinal microbiota, increased gut hormone concentration, influenced proteolysis, restoring impaired proteasome activities and modulating the autophagic flux [142]. SLAB51 treatment also reduced oxidative stress in AD mice brain via sirtuin 1-dependent mechanisms [143]. Attenuation of learning and memory deficits and recovery of Aβ and Tau protein levels as well as cytokine levels in blood were reported after administration for four weeks of oligosaccharide extracted from *Morinda officinalis* to D-galactose evoked AD-like symptoms in rats [144]. The role of FMT in AD has been recently investigated in APPswe/PS1dE9 transgenic mouse and in a novel animal model of AD, the ADLP^APT^ mice characterized by three human transgenes, including amyloid precursor protein, presenilin-1, and tau which shows amyloid plaques, neurofibrillary tangles and reactive gliosis [145,146,147]. In an APPswe/PS1dE9 transgenic mouse model, FMT attenuated cognitive deficits and synaptic disfunction as well as reduced the neuroinflammatory markers [145]. In addition, FMT from WT mice into ADLP^APT^ mice ameliorated the formation of Aβ plaques and tau, glial reactivity and cognitive impairment. Moreover, FMT, restored intestinal macrophage activity and inflammatory blood monocyte population altered in transgenic mice [147]. In AD patients, treatment with *Lactobacillus acidophilus*, *Lactobacillus casei*, *Lactobacillus fermentum*, and *Bifidobacterium bifidum* for three months improved cognitive functions and metabolic status as the probiotic treated group exhibited better Mini-Mental State Examination (MMSE) score and reduced markers of insulin metabolism, and serum levels of triglyceride and Very Low Density Lipoprotein (VLDL) [148]. Multispecies probiotic (*Lactobacillus casei* W56, *Lactococcus lactis* W19, *Lactobacillus acidophilus* W22, *Bifidobacterium lactis* W52, *Lactobacillus paracasei* W20, *Lactobacillus plantarum* W62, *Bifidobacterium lactis* W51, *Bifidobacterium bifidum* W23 and *Lactobacillus salivarius* W24) supplementation might modify gut bacteria composition and tryptophan metabolism of AD patients as an increase in *Faecalibacterium prausnitzii* with enhanced serum kynurenine concentrations were observed after 28 days of treatment [149]. Finally, a recent systematic review and meta-analysis of randomized clinical trials reported that evidence to support the clinical application of probiotics to improve cognitive function in humans with dementia is insufficient [150].

**Table 1:** Effect of probiotic supplementations in AD-like animal models and human patients.

Model System	Probiotic Supplementation	Pathological Signature	Reference
amyloid (1–42) injected rats	*L. acidophilus*, *L. fermentum*, *B. lactis*, and *B. longum*	Prevented learning and memory impairment and decreased the number and size of plaques	[140]
amyloid (1–42) injected rats	*L. acidophilus*, *B. bifidum* and *B. longum*	Restored the hippocampus dependent spatial memory and synaptic plasticity damaged	[141]
3xTg-AD transgenic mice	SLAB51	Counteracted cognitive decline and brain damage, increased gut hormone concentration, restored impaired proteasome activities, modulated the autophagic flux and reduced oxidative stress	[142,143]
D-galactose treated rats	oligosaccharide extracted from *Morinda officinalis*	Attenuated learning and memory deficits, increased antioxidant activity and acetylcholine levels	[144]
AD patients	*L. acidophilus*, *L. casei*, *L. fermentum*, and *B. bifidum*	Improved cognitive functions and metabolic status, reduced markers of insulin metabolism and serum levels of triglyceride and VLDL	[148]
AD patients	*L. casei* W56, *L. lactis* W19, *L. acidophilus* W22, *B. lactis* W52, *L. paracasei* W20, *L. plantarum* W62, *B. lactis* W51, *B. bifidum* W23 and *L. salivarius* W24	Enhanced serum kynurenine concentrations	[149]

## 5. Crosstalk between Gut Microbiota and eCB System

The eCB system is widely distributed throughout the gut in health and disease and its involvement in gastrointestinal physiological and pathophysiological processes has been described [151,152]. As the microbiota is the main character in the intestinal tract, alterations in the gut microbiota composition might influence the eCB system signaling or they might influence each other while performing their role in the gut. Studies that were mainly focused on metabolic and obesity-related disorders have suggested that modulation of the eCBome is associated with changes in the gut bacterial community and, on the other hand, the modification of the gut microbiota by using probiotics, antibiotics or GF mice affected eCB signaling.

In a mouse model of diet-induced obesity (DIO), CB_1_ antagonist treatment increased *Akkermansia muciniphila* and decreased *Lanchnospiraceae* and *Erysipelotrichaceae* levels in the gut, attenuated inflammatory state and improved hyperglycemia and insulin resistance [153] while THC chronically administered in mice prevented DIO-induced increase of the *Firmicutes:Bacteroidetes* ratio [154]. Mice lacking the *Napepld* gene in their adipose tissue increased body-weight gain, insulin resistance, adipose tissue inflammation and altered lipid metabolism. In addition, the mutant mice exhibited lower levels of NAEs and a shift in gut microbiota composition as decreased levels of *Lactobacillus* and *Allobaculum* genera were observed [155]. Mice lacking the *Mgll* gene were protected against high-fat diet (HFD)-induced obesity, insulin resistance and hepatic steatosis [156] and exhibited significantly higher levels of *Hydrogenoanaerobacterium*, *Roseburia*, and *Ruminococcus* [157]. Reduction of colonic CB_1_ mRNA expression accompanied by increased expression of FAAH and reduction of AEA levels were reported in a genetic model of obesity, the *ob*/*ob* mice, treated with prebiotics for 5 weeks. Still, CB_1_ expression levels were also reduced in lean wild-type mice after antibiotic treatment for 2 weeks [158]. Furthermore, mice treated for 7 days with non-absorbable-broad spectrum antibiotics selectively upregulated the expression of CB_2_ and exhibited altered microbiota profile as luminal counts of *Lactobacillus* and *Enterobacteria* were increased whereas the *Clostridium* and the *Verrucobacteria* groups were reduced [159]. *Lactobacillus acidophilus* treatment increased the expression of CB_2_ and induced analgesia in a rat model of chronic colonic hypersensitivity [160]. The cause-effect relationship between altered microbiome and modulation of eCBome were explored by assessing eCBome gene expression levels in small intestine of young and adult GF mice as well as in GF mice after microbiota re-introduction by FMT procedure [161]. In 13 weeks-old GF mice upregulation of CB_1_ and PPARα and downregulation of GPR18 and GPR55 expression were reported, while genes encoding for NAE synthesis significantly increased in both 4- and 13-weeks-old GF mice. Notably, these modifications were, partially or completely, reverted after FMT from donor to age-matched GF mice [161]. Recently, in a mouse model of depression, the unpredictable chronic mild stress (UCMS) model, Chevalier and coworkers correlated the effect of gut microbiota on depressive-like behaviors in mice with modulation of the eCB system signaling. The authors reported that naive unstressed mice that underwent the FMT procedure from UCMS donors developed depressive behavioral symptoms and exhibited reduced neurogenesis in the hippocampus accompanied with a significant decrease of 2-AG levels in both UCMS donors and recipients. Of note, pharmacological modulation of 2-AG signaling, by using MAGL inhibitor, or complementation with a *Lactobacillus* probiotic strain normalized depressive symptoms and neurogenesis in recipient mice [162]. In a GI colonization model, mice inoculated with commensal fungus *Candida albicans* altered eCB levels in the brain and GI tract, increased anxiety-like behavior and basal levels of the stress hormone corticosterone. Treatment with an FAAH inhibitor, URB597, reverted both neuroendocrine phenotypes [22].

Antibiotic-induced experimental dysbiosis in mice caused social behavior alterations and depressive-like behavior, significant decreased brain-derived neurotrophic factor (BDNF) expression and increased phosphorylation, and hence increased sensitization, of TRPV1 in the hippocampus [21]. These alterations were counteracted by subsequent probiotic administration. In addition, intestinal levels of *N*-arachidonoylserotonin and *N*-oleoylserotonin, two members of the eCBome, decreased in dysbiotic mice and increased after probiotic treatment in the mouse jejunum [21].

Few human studies have been reported so far on the linking changes in gut microbiota composition with changes in eCB system mediators and proteins. In a randomized clinical trial including 60 obese people, OEA supplementation, for 8 weeks, significantly decreased carbohydrate intake and increased the abundance of *Akkermansia muciniphila* bacterium, one of the next-generation beneficial microbes, inversely associated with obesity and diabetes [163,164]. In addition, in a cohort consisting of 32 overweight or obese subjects, 3 months of daily supplementation with *Akkermansia muciniphila* exerted its beneficial effects in a way independent from the modulation of the plasmatic levels of 25 different eCBome-related lipids [165]. *Akkermansia muciniphila* supplementation prevented the reduction of 2-palmitoylglycerol (2-PG) levels in human patients and increased production of 2-PG, 2-oleoylglycerol (2-OG), and 2-AG in obese mice [165,166]. Recently, by using a multilevel mediation model that establish alpha diversity, within individual gut-microbial diversity, as predictor, serum and fecal levels of PEA as mediator, and anhedonia/amotivation as outcome, PEA was reported to mediate the association between gut-microbial diversity and anhedonia/amotivation in a longitudinal study performed on 786 volunteer twins [167]. Finally, in a heterogeneous human population of 195 healthy volunteers, changes in circulating levels of MAGs and NAEs were correlated with diet-induced changes in gut microbiota composition [168]. In particular, NAE levels were enhanced in elevated fat mass volunteers, while 2-MAGs were increased in individuals with predominant visceral body fat distribution. Subject that self-reported higher omega-3 fatty acid intakes exhibited higher level of omega-3 derived NAEs and 2-MAGs and, while several NAEs were positively associated with *Peptostreptoccocaceae* and *Veillonellaceae* families, 2-MAG levels were negatively associated with *kermansiaceae* [168].

## 6. The eCBome as the “Hidden” Player between the Gut and the Brain in AD

Studies most in the context of metabolic dysfunctions, already reviewed elsewhere [19,169], suggest cross-talk between the gut microbiome and the eCBome. However, the connection of the gut-brain axis and the eCBome in neurodegenerative diseases has never been deeply investigated but, considering the important role of these two systems in the physiopathology of the CNS, it is worth doing it. As already stated above, even if no specific data exist in the literature yet, we will try to elucidate some possible mechanisms that underline the communication between the gut-brain axis and the eCBome in the AD.

In the last decades the modulation of the eCB system has emerged as a potentially attractive therapeutic strategy for the treatment of AD (Figure 1), as reviewed in [17,18,170]. In particular, human studies have shown that CB_2_ receptors and FAAH are overexpressed in neuritic plaque-associated glia analyzed in post-mortem AD brains [171], especially in perivascular microglial cells [172]. Moreover, the expression of CB_2_ and FAAH is related to the Aβ deposition suggesting a possible regulatory role associated with the pathological alterations of AD induced in microglial cells. On the other hand, the role of CB_1_ is debating, but human studies have pointed out that CB_1_ activity is higher at earlier AD stages with a later decrease [173]. Furthermore, in an in vitro study, CB_1_ activation has revealed the beneficial neuroprotective effects reducing Aβ deposition and tau phosphorylation [174]. The question is, how does the eCBome communicate with the microbiome gut-brain axis and what is the role of this interplay in the pathophysiology of the AD?

We consider two hypotheses to answer to this: neuroinflammation and obesity as risk factor for AD. Neuroinflammation is related to microbial translocation by a leaky gut barrier which in turn may affect the CNS through the VN and enteroendocrine signaling [175]. In this context, it is worth mentioning the well-established LPS translocation pathway and chronic inflammation in the CNS due to microglial activation that could be modulated by eCBs. In fact, AEA can induce gut permeability, causing a ‘leaky’ gut which in turn lead to the onset of metabolic endotoxemia releasing toxins, including LPS, to cross the epithelial barrier driving inflammatory signaling and affecting the CNS [20]. In particular, primarily by leading the release of pro-inflammatory cytokines and other neurotrophic factors from the mesenteric lymph nodes which albeit to BBB disruption, LPS favors the infiltration of leukocytes into the CNS and finally promotes the development of neuroinflammation state. Therefore, targeting the eCB system in the gut can modulate the integrity of intestinal barrier [176]. Furthermore, leaky gut and endotoxemia are characteristic features of obesity [177] that is considered a risk factor for AD. In recent years, the link between the obesity and the subsequent leptin and insulin impaired signals with the onset of the AD pathology have been studied [178]. In particular, the increased adipose tissue in obesity could trigger a blood flow decrease to brain, which lead ischemia in vulnerable areas, such as neurons in the hippocampal regions CA1, CA3, and CA4, that in turn could be one of the main cause of increased memory loss [179,180]. Moreover, the release of adipose tissue hormones, adipokines as leptin and other cytokines causes chronic inflammation in the periphery that may reach the CNS leading to neuroinflammation, reduction of brain white matter and impairment of neuronal connections [181,182]. Several studies supported the connection between leptin levels and AD pathophysiology [183] as leptin levels were found to be lower in AD patients than in healthy controls, suggesting a positive correlation with the reduction of AD risk [184]. Furthermore, chronic leptin administration improved memory functions and Aß clearance in a transgenic animal model of AD [185]. Of note, leptin and eCBs are strictly correlated, as first demonstrated by Di Marzo et al. [186], showing that leptin inhibits the biosynthesis of eCBs in the hypothalamus in the appetite-related circuit via orexigenic mediators. Moreover, in mice leptin is able to decrease the release of eCBs by inhibiting voltage-gated calcium entry [187]. Therefore, it is likely to suggest that the interplay between eCBs and leptin is one of the not well-investigated mechanisms through which the gut-brain axis is regulated under pathological conditions, especially in the context of AD. As mentioned in the previous sections, elevated tissue levels of 2-AG in *Mgll*^−/−^ mice are associated with resistance to the metabolic alterations induced by a HFD as they accumulated less fat and became less glucose intolerant and insulin resistant than WT mice following HFD [156] and changes in *Mgll*^−/−^ mice microbiome have been reported to contribute to their obesity resistant phenotype [156,157]. In addition, pharmacological elevation of 2-AG levels with MAGL inhibitor prevented neuroinflammation and decreased neurodegeneration in different AD-like animal models [188,189]. Of note, deletion of MAGL in astrocytes attenuated LPS-induced neuroinflammation in mice and genetic MAGL inactivation in PS1/APP AD model reduced prostaglandin production, Aβ levels and plaques [190,191]. This suggests that MAGL is a key modulator of gut microbiota composition, inflammation and amyloidosis and might be considered as a potential next-generation target whose deep investigation might provide new therapeutic strategy against AD etiology and its modifiable risk factors.

## 7. Conclusions

Although both the eCB system and the gut microbiota have individually emerged as molecular targets in the pathology of AD as they may counteract inflammatory, neurodegenerative and cognitive aspects of the disease, research on the complex interactions of these systems in AD is still missing.

The overlapping roles of the eCB system and the microbiome in many diseases including dysmetabolism, obesity and neuropsychiatric disorders [19,44] suggest that a novel approach such as modulating the microbiota via eCB system may provide new therapeutic perspectives for treating AD. In particular, therapeutic strategies derived by diets or prebiotic and probiotic supplementation that might promote and support the growth of bacteria synthetizing beneficial mediators, eCBs and eCB-like compounds included, acting as AD-modifying drugs should be examined. On the other hand, the potential beneficial role of increased levels of 2-AG and hence the activation of CB_2_ and other molecular targets belonging to the family of eCBome receptors mediating inflammation and gut microbiota composition and diversity should be clarified.

In conclusion, studies are now needed to provide answers to the question of whether or not the eCB system can be considered a bridge between gut microbiota and AD to be target for the development of applicable interventions for the treatment of the progress of neurodegenerative disorders.

## Data Availability

Not applicable.
